# The changing climates of global health

**DOI:** 10.1136/bmjgh-2021-005442

**Published:** 2021-03-23

**Authors:** Thomas Cousins, Michelle Pentecost, Alexandra Alvergne, Clare Chandler, Simukai Chigudu, Clare Herrick, Ann Kelly, Sabina Leonelli, Javier Lezaun, Jamie Lorimer, David Reubi, Sharifah Sekalala

**Affiliations:** 1 Institute of Social and Cultural Anthropology, University of Oxford School of Anthropology and Museum Ethnography, Oxford, Oxfordshire, UK; 2 Department of Sociology and Social Anthropology, University of Stellenbosch, Stellenbosch, South Africa; 3 Global Health and Social Medicine, King's College London, London, UK; 4 SAMRC-Wits Developmental Pathways for Health Research Unit, School of Clinical Medicine, University of the Witwatersrand, Johannesburg-Braamfontein, South Africa; 5 Montpellier Institute of Evolutionary Sciences, University of Montpellier, Montpellier, Languedoc-Roussillon, France; 6 Institute of Human Sciences, University of Oxford School of Anthropology and Museum Ethnography, Oxford, Oxfordshire, UK; 7 Department of Global Health and Development, London School of Hygiene and Tropical Medicine Faculty of Public Health and Policy, London, UK; 8 Oxford Department of International Development, University of Oxford, Oxford, UK; 9 Geography, King's College London School of Social Science and Public Policy, London, UK; 10 Sociology, Philosophy and Anthropology, University of Exeter College of Social Sciences and International Studies, Exeter, Devon, UK; 11 Institute for Science, Innovation and Society, University of Oxford School of Anthropology and Museum Ethnography, Oxford, Oxfordshire, UK; 12 School of Geography and the Environment, University of Oxford Social Sciences Division, Oxford, Oxfordshire, UK; 13 Global Health and Social Medicine, King's College London School of Social Science and Public Policy, London, UK; 14 School of Law, University of Warwick Faculty of Social Sciences, Coventry, West Midlands, UK

**Keywords:** COVID-19, health policies and all other topics, environmental health

**Summary box**

- The historical trajectories of three crises have converged in the 2020s: the COVID-19 pandemic, rising inequality and the climate crisis.
- Global health as an organising logic is being transformed by the COVID-19 pandemic.
- We point to an emerging consensus that the triple threats of global heating, zoonoses and worsening, often racialised inequalities, will need to be met by models of cooperation, equitable partnership and accountability that do not sustain exploitative logic of economic growth.
- Health governance is challenged to reconsider sustainability and justice in terms of how local and global, domestic and transnational, chronic and infectious, human and non-human are interdependent.
- In this article, we discuss their intersection and suggest that a new set of organising ideals, institutions and norms will need to emerge from their conjunction if a just and liveable world is to remain a possibility for humans and their cohabitants.
- Future health governance will need to integrate pandemic preparedness, racial justice, inequality and more-than-human life in a new architecture of global health.
- Such an agenda might be premised on solidarities that reach across national, class, spatial and species divisions, acknowledge historical debts and affirm mutual interdependencies.

## Introduction

The decisions we make now will determine the course of the next 30 years and beyond: Emissions must fall by half by 2030 and reach net-zero emissions no later than 2050 to reach the 1.5C goal…If we fail to meet these goals, the disruption to economies, societies and people caused by COVID-19 will pale in comparison to what the climate crisis holds in store. (António Guterres, United Nations Secretary-General)^1^

The historical trajectories of three crises have converged in the 2020s: the COVID-19 pandemic, rising inequality and the climate crisis. The political, social and institutional arrangements that have collectively constituted 'global health,' and the potential obstacles and possibilities of the COVID-19 pandemic reveal the intersecting challenges of rising inequality and climate crisis. Emerging transformations in global health, accelerated by the sea changes of the 2020s, are characterised by attempts to expand notions of health and social justice encompassing planetary, racial, reproductive and digital justice. In this article, we discuss their intersection and suggest that a new set of organising ideals, institutions and norms will need to emerge from their conjunction if a just and liveable world is to remain a possibility for humans and their cohabitants.

## The end of global health?

'Global health' has been a contested project since its emergence as a framework and field of practice over 20 years ago.^2 3^ Variously conceived of as either ‘the health of populations in a global context’ or the particular assemblages of institutions, practices, technologies and norms that produce a field of concern called ‘global health,’ it has remained an unstable object of analysis.^4^ Critical global health scholarship, in particular, has attended closely to the configurations of knowledge and power that have defined global health as a political enterprise, scrutinising the forms of evidence, efficacy and accountability that are at work in its programmes and tracing the uneven delivery of its promises.^4–8^

The shift from international health to global health in the 1990s indexed changing geopolitical arrangements. It embodied a growing understanding that health transcends national boundaries and actors, and that chronic inequities were rooted in transnational determinants (war, climate, neoliberalism) that only large-scale holistic perspectives could address. One criticism has been that instead of levelling the playing field, global health has reinscribed colonial power differentials, as discourses, technologies and priorities tend to be set by agencies located in high-income countries (HICs) for export to the rest of the world.^9^ The ‘global’ in global health has in practice referred to the reach of a small set of non-state actors—non-governmental organisations, pharmaceutical companies, philanthropies and universities—capable of defining new health agendas for the planet.

The peculiar epistemology of global health emerged in the post-Cold War period from the widely shared belief that the transnational nature of contemporary threats to health—the propagation of infections through air travel, or the rise of chronic diseases associated with trade liberalisation and multinational corporations—could not be addressed through the old international health system, built around nation-states, but required new global solutions able to work across political and geographical borders.^10–14^ These global solutions included novel funding mechanisms like the Global Fund to Fight AIDS, Tuberculosis and Malaria, supranational regulatory arrangements like the WHO’s Framework Convention on Tobacco Control and infrastructures allowing medical commodities to travel more efficiently across borders.^3^ Influenced by the then dominant neoliberal view of the state, efforts to improve health focused on the role of non-state actors, such as civil society groups, private–public partnerships and philanthropies. This was also an era characterised by a preference for portable micro-technologies—such as bed nets or point-of-care diagnostics—and pharmaceutical commodities over investments in national health infrastructures or efforts to transform political and economic systems.^5 15–18^ The successes associated with these newly global arrangements have nevertheless been significant, from malaria control (global rates fell by 60% between 2000 and 2015, although progress since has stalled) to the eradication of polio in Africa in 2020.

Yet the utility of ‘global health’ as a framework has come under increasing strain over the last decade, under the weight of shifting geopolitical interests, worsening inequalities and the intensifying urgency of the climate crisis. Well before the COVID-19 crisis, the response to the HIV, Ebola or Zika epidemics showed that ‘therapeutic geographies’ continued to be profoundly shaped by histories of race, colonial legacies and postcolonial geopolitics.^19–21^ Long-standing discontent over the exclusion of expertise from the global South, the growing bifurcation between those providing and those receiving global health assistance, and the racialised nature of access to care in the global North have also come into sharper focus.^22–25^ In the meantime, new geopolitical cleavages, growing protectionism, the sidelining of the climate emergency, and the dissemination of powerful but unevenly impactful digital technologies are changing the meaning of ‘global’ and ‘health’ in rapid and at times unpredictable ways.

The COVID-19 response has thus far been characterised by a reinforcement of some aspects of the global health episteme, such as the focus on biomedical solutions, philanthropic models of pharmaceutical delivery, and measurable and cost-effective interventions. As historian Thomas Laqueur^26^ remarks, many people have experienced the COVID-19 pandemic through models and numbers developed and disseminated by epidemiologists, computer scientists, modellers and mathematicians. These are the numbers and models that governments across the world use to govern and control the epidemic, building on recent developments in data science, surveillance technologies and artificial intelligence.^27^ The potential for digital technologies to revolutionise public health has also come into sharp focus, with many governments investing in mass digital health surveillance and artificial intelligence solutions despite the dramatic differences in individual access to digital services and measures for data protection.

Conversely, other aspects of global health are being challenged.^28^ National rather than global solutions have been at the forefront of the response. This is most obvious in relation to quarantine measures and lockdowns, with nation-states closing their borders, freezing their economies and limiting their citizens’ freedoms in previously unimaginable ways.^29^ It can also be seen in countries’ efforts to strengthen and expand their healthcare systems and capacities, redefining medical research infrastructures as matters of national security. The virtues of off-shoring industrial production have been put into question, as some countries seek to renationalise the production of masks, hand-sanitisers, ventilators and vaccines.^30^ ‘Vaccine nationalism’ remains a present threat to the ability to expand access across the world.^31^

Furthermore, trust in science and reason, foundational to global health and its formal commitment to ‘evidence-based’ interventions, has become precarious and contested. In liberal democracies, questions about the provenance and validity of scientific data have dominated debates over national lockdowns, and there has been significant backlash against individual experts or expert advisory bodies associated with the imposition of emergency measures. For example, Bill Gates, a key figure of global health, has become the target of conspiracy theories about the role of COVID-19 as the instrument of a new ‘world order’.^32 33^ In the wake of the proliferation of conspiracy theories about COVID-19 and their potential impact on vaccination and containment strategies, ‘infodemiology’ has emerged as a community of practice and research premised on the apparent infectiousness of information in the age of globalised social media. As a new chapter in health communication, infodemiology indexes a longer history of the mystification of global health governance for the lay public, including obscure patent laws, questionable metrics and opaque structures of accountability. Such tensions manifest in the material sites of basic health systems; drugs and diagnostics (access, certification, regulation, infrastructure, distribution); data management, analytics and surveillance mechanisms; and the competing knowledge and expertise that inform global health governance. New tensions have also arisen between social justice as a core value for many global health workers who have been forced to ration scarce resources and a renewed call for solidarity to respond to the pandemic, racism and the climate crisis.^34^ These values will come under even greater pressure as questions about the equity of vaccine distribution accelerate.

The rise and fall of ‘global health’ as a dominant set of policies and arrangements also tracks the rise (and arguably the failure) of planetary concerns with climate, biodiversity and sustainability. Global health has not (yet) departed from the anthropocentrism that lies at the root of these interlinked planetary crises ranging from: global heating and extreme weather to land use change and biodiversity loss to the threats posed by intensive animal agriculture as a driver of zoonotic disease, a crucial factor in preventing future pandemics.^35^

What these structural dynamics suggest is a need to re-examine how scalar differences between domains of action (eg, local/global) and degrees of concern (eg, the category of ‘neglected disease’) are produced and re-produced by the institutions of global health governance, and what alternative configurations might better address our contemporary world. In this context, the ongoing COVID-19 crisis is producing new assemblages of expertise and modes of intervention. The uneven impact of the coronavirus pandemic has been a reminder of the difference between contexts where strong public health systems exist (eg, New Zealand, Germany, Vietnam) and those where public health has been eroded by austerity and neoliberal approaches to healthcare (eg, UK, USA, Brazil). While the pandemic has given the lie to any self-evident divide between North and South, it has also reignited calls to reckon with the colonial and exclusionary legacies that are constitutive of the global health enterprise.^36^

## The new syndemics

Global health institutions, norms, policy and practices have historically been configured by a prioritisation of the ‘big three’ (malaria, HIV and tuberculosis (TB)). Since the outbreak of the COVID-19 pandemic, some have decried the ‘COVIDisation’ of research,^37^ and of global health more generally, prompting concern from scholars and practitioners for what is becoming a series of knock-on crises for maternal and child health, HIV, malaria, TB, cancer, fertility treatment, elective orthopaedics, among others. In its 2020 *Goalkeepers Report,* the Gates Foundation expressed concern that its focus on the ‘residual pandemics’ that still cause significant mortality and morbidity in low/middle-income countries (LMICs) was facing an existential threat from COVID-19. Such concern ironically recapitulates earlier critiques of global health for the way in which it ‘siloed’ health programming while displacing attention to the structural determinants of health.^4^ In addition to the enduring health crises that have animated global health, COVID-19 has produced new syndemics and categories of vulnerability, particularly around malnutrition (including hunger and obesity), even while these map onto long-existing structures of inequality.

COVID-19 has rendered visible health categories that had previously only occupied those in public health or medicine. In the process, these categories have also been mainstreamed as part of the pandemic’s explanatory lexicon. Politicians and the public now equally talk of ‘pre-existing’ conditions—a flexible shorthand for a variety of chronic, non-communicable diseases (NCDs)—that are used to mark out and explain risk and vulnerability to ‘serious illness’ should an individual contract COVID-19.^38^ The category of NCDs has long stood in contrast to those diseases spread by pathogens, rather than *pathogenic conditions*. Yet we have been repeatedly warned that those same pathogenic living conditions—urbanisation, inequality, globalisation, intensive agriculture and environmental degradation—that were understood to drive the risk of NCDs are also those that enable the emergence of novel infectious diseases.^39 40^ With NCDs modulating the risk of morbidity and mortality for COVID-19 and the disease itself creating long-term, chronic sequelae, the distinction between categories of communicable and non-communicable is increasingly porous. As some have thus noted, COVID-19 is not simply a viral infection, but rather a complex syndemic with clinical and structural vulnerabilities entrenched by existing poor health, employment and precarity, deprivation and inequalities.^38 41 42^ As the categories, crises and scaffolding of the global health enterprise transform, new questions are emerging of how to reconceptualise the architecture of global health.

Before the advent of HIV/AIDS, Stephen Morse argued that, ‘it had become commonplace to suggest that infectious diseases were about to become a thing of the past and that chronic, non-infectious diseases should be our major priorities’.^39^ The notion of the ‘epidemiological transition’ has not borne out for developed or developing contexts,^43 44^ as the COVID-19 crisis makes plain. The ‘smug sense of immunity from infectious diseases that characterised health professionals in North America and Europe’^45^ has given way to a new emerging infectious disease paradigm, characterised by preparedness and simulation.^46^ COVID-19 has revealed the lack of pandemic preparedness and complacency about emergent infectious disease in Europe and the USA, and in many cases the chronic underfunding of public systems that were previously robust. It has also called into question the ways in which pandemic preparedness is measured and understood in high-income settings.^30 47^

Despite Morse’s fear that the chronic has been prioritised over the infectious, funding for NCDs within the complex ecosystem of global health has consistently remained below 2% of total disbursements.^48^ While national health policies in the global North may have side-lined the ever-present threat of epidemic possibility, the spectre of infectious disease threats including in the form of drug-resistant infections has loomed in recent years.^49^ The global nature of these threats has been articulated in the rhetoric of health security, recognising the danger emergent from global health ‘hotspots’, particularly in sub-Saharan Africa. Here, international donor money and priorities have largely dictated domestic health agendas, regardless of the particularity of biomedical need.^50^ Indeed, in almost all countries, rising rates of chronic disease coexist with actual and potential infectious disease threat. The problem then is not really whether infectious diseases were deprioritised or not, but rather that the unwillingness to prioritise chronic diseases within global health has consistently undermined efforts to address the broad political, economic, social and environmental *determinants* of health conceptualised in the broadest possible sense. Despite 40 years of appeals to pay attention to ‘structural determinants’, the social determinants of health remain a sanitised framework that risks overlooking health justice as a key component of achieving health for all.^51^

Perhaps most importantly, the pandemic has laid bare the *global* lack of adequate attention to the upstream drivers of health within the global health enterprise. Global health has largely failed either to sustain much interest in, or to have any concrete impact on, the conditions that create vulnerability to disease exposure, the likelihood of serious illness or the possibility of recovery.

A key example is the efforts to link human and animal health within a global governance regime of one health or planetary health over the last decade. It has proven difficult to join up zoonotic disease, biodiversity loss, and climate change into either a meaningful global political order or workable global health endeavour.^52^ The COVID-19 pandemic brings these interdependencies sharply into focus: while the specific origins of COVID-19 remain uncertain, it has become clear that the pandemic is a consequence of the intensive and globalised nature of the contemporary food system.^53^ The virus emerged in an agricultural setting and spread through global networks of trade, tourism and commerce. COVID-19 has generated an amplified awareness of the risks of zoonotic disease and of the latent potential of new epidemics due to land use change and industrial agriculture. It also reveals a lack of appreciation of evolutionary ecology and host–pathogen coevolution. Rather than militarised vocabularies of an ‘arms race’ against microbes which sees disease eradication as the goal, a more dynamic understanding would frame health as adaptation rather than absolutist concepts of health with humans positioned as sovereign agents.

## The return to law as usual

Global health as an organising logic has depended on particular arrangements of institutions, policies, norms and instruments. The development of international law and global health governance has been a key factor in the functioning of these systems. Even before the COVID-19 crisis, it was difficult to gain consensus on global health law.^54^ Many new institutions and funders were unilaterally engaged in making ‘soft’ laws that often focused on single issues at the expense of collective decision-making.^55^ The legal landscape for infectious diseases was fragmented and crowded with multiple competing actors and regimes that covered many areas such as health security, border control, surveillance, trade and intellectual property.^56^ This fragmentation has made it difficult to create cohesive legal regimes that recognise the impacts of planetary degradation on health outcomes. For example, attempts to link global health law to planetary health through instruments such as the Paris Agreement have established weak obligations on states and thus have not translated into cohesive action.

The COVID-19 pandemic has however forced a renewed recognition of the centrality of law in declaring and managing health crises. States have used law strategically to gain better health outcomes for citizens and to push nationalist agendas.^57^ Many countries have passed emergency regulations with little or no scrutiny.^58^ At the international level, both states and international actors have expressed heightened interest in the International Health Regulations (IHR) 2005, which are the sole binding global legal instrument dedicated to the prevention and control of the international spread of infectious disease. While these rules have had some effect, they have also been strategic tools with performative value, allowing some states to ignore obligations under the IHR while pressuring the WHO to act on other issues. For instance, although many states created emergency laws in order to close borders, on the basis of Article 43 of the IHR, these border closures were far from universal. The WHO has also been accused of declaring the COVID-19 crisis a Public Health Emergency of International Concern—its highest alert level—too late, yet at the same time, the majority of states have ignored the WHO’s advice on a wide range of issues from border closures, contact tracing, vaccine nationalism to stockpiling products at the expense of developing countries.^59 60^

The COVID-19 pandemic has also given the lie to the notion that good global health governance could ensure just and equitable distribution by means of law alone. For example, the distribution of vaccines is predicated on countries’ abilities to purchase supplies, leaving developing countries at the back of the queue. Attempts by developing countries to relax World Trade Organization rules so that LMICs could create their own vaccines have faced resistance from HICs and Big Pharma. In the face of a truly global crisis, it is clear that revolutionising the law to create greater access to essential medicines is extremely difficult. Instead, the law frames the crisis as an exception and reinstates existing inequities in the political economy of access to health.

## Conclusion

The COVID-19 pandemic has shone a harsh light on the fractures and limitations of the global health apparatus, forcing a generalised rethink of institutional arrangements and modes of action. Whatever governance regimes for health and climate emerge from the current crises, we will need a new, and better, concept of ‘the global’, even as states reinvent the social compacts that have historically shaped claims to rights and protections in particular national contexts. As consensus emerges on the triple threats of global heating, zoonoses and worsening, racialised inequalities, new models of cooperation, equitable partnership and accountability that avoid exploitative logics of economic growth will be needed.^61^ ^62^ There is increased advocacy for a just model of health that recognises the shared suffering of diverse human and non-human actors, while acknowledging the claims for a healthy future made by generations of diverse life forms.^63^ The concept of planetary justice is gaining traction across various domains and could prove a vital framework for a new, reimagined global health agenda.^64–66^ Such an agenda might be premised on solidarities that reach across national, class, spatial and species divisions, acknowledge historical debts and affirm mutual interdependencies.^67–69^ As WHO Director-General Tedros Ghebreyesus has said, “None of us are safe until all of us are safe.”
